# Acute Respiratory Distress Syndrome Caused by Varicella Pneumonia in an Immunocompetent Adult: A Case Report

**DOI:** 10.7759/cureus.94042

**Published:** 2025-10-07

**Authors:** Mohammed K Zubaidi, Haneen Hussein Sahib, Younus M Al-Khazaali, Moamel Jaafar Ali, Ali J Ahmed

**Affiliations:** 1 Internal Medicine, College of Medicine, Al-Nahrain University, Baghdad, IRQ; 2 Surgery, College of Medicine, Al-Nahrain University, Baghdad, IRQ

**Keywords:** acute respiratory distress syndrome, ards, pneumonia, pulmonology, varicella pneumonia, varicella-zoster virus, vzv

## Abstract

Varicella infection, caused by the varicella-zoster virus (VZV), is a self-limiting disease in immunocompetent individuals. However, in patients with immunosuppression, underlying pulmonary disease, and risk factors such as aging, smoking, and pregnancy, it can lead to a potentially life-threatening disseminated disease. Lungs are the most frequently affected internal organ in disseminated VZV. Varicella pneumonia commonly presents with progressive respiratory symptoms accompanied by a cutaneous rash. Varicella pneumonia can rapidly progress to acute respiratory distress syndrome (ARDS). Timely clinical recognition, supported by radiological findings and molecular testing, along with early treatment, is key to improving prognosis. We present a case of ARDS complicating varicella pneumonia in an immunocompetent 25-year-old male who presented with skin rash and respiratory compromise, but without inflammatory nodules on chest imaging.

## Introduction

Varicella infection, caused by the varicella-zoster virus (VZV), is responsible for the following clinical syndromes: varicella (chickenpox), herpes zoster (shingles), and disseminated disease [1]. In immunocompetent adults, VZV infection is usually benign. However, immunocompromised adults and patients with risk factors such as underlying pulmonary disease, aging, smoking, and pregnancy are at increased risk for disseminated diseases such as viral pneumonitis, hepatitis, pancreatitis, myocarditis, and nephritis that carry significant morbidity and mortality [2].

Lungs are the most frequently affected internal organ in disseminated VZV. Although mostly affecting immunocompromised patients, varicella pneumonia can also lead to severe and sometimes fatal outcomes in immunocompetent individuals. Varicella pneumonia presents with shortness of breath, cough, pleuritic chest pain, hemoptysis, and fever, accompanied by a vesicular skin rash [3]. We report a case of acute respiratory distress syndrome (ARDS) related to varicella pneumonia in an immunocompetent 25-year-old male who presented with diffuse skin rash and dyspnea, in the absence of characteristic inflammatory nodules on chest imaging.

## Case presentation

A 25-year-old male with an unremarkable past medical history presented to the emergency department with progressive dyspnea over the past two days. Five days before his presentation, the patient had a fever and generalized skin rash that started on his trunk and soon spread to his face and all extremities associated with pruritus. These symptoms were followed by a dry cough and one episode of scanty hemoptysis four days before hospital admission. The patient was initially treated at home with acetaminophen, diclofenac, and chlorpheniramine with minimal relief of his symptoms. The patient reported no sick contacts and an unknown vaccination status. The patient was a non-smoker with no history of illicit drug use. 

The physical examination on admission was notable for a confused young male with acute distress and increased work of breathing. His vital signs were as follows: fever at 39°C, tachypnea at 31 cycles/minute, blood pressure of 115/76 mmHg, heart rate of 130 beats/min, and pulse oximetry at 76% on oxygen supplementation via a non-rebreather mask at 15 L/min. On chest examination, there were bilateral crackles in the upper and lower lung fields. He had a generalized papulovesicular rash with crusts on his face, trunk, and all extremities. Initial laboratory investigations demonstrated leukocytosis, thrombocytopenia, hyponatremia, normal anion gap metabolic acidosis, transaminitis, and elevated C-reactive protein (CRP). Laboratory tests, including arterial blood gas (ABG), are summarized in Table 1. At a fractional inspired oxygen (FiO_2_) of 0.6 with an arterial oxygen partial pressure (PaO_2_) of 74 mmHg, the resulting PaO_2_/FiO_2_ ratio of 123 mmHg indicates moderate hypoxemia.

**Table 1:** Laboratory findings on admission. MCV: mean corpuscular volume; AST: aspartate aminotransferase; ALT: alanine aminotransferase; PaO₂: arterial partial pressure of oxygen; PaCO₂: arterial partial pressure of carbon dioxide; HCO₃⁻: bicarbonate; CRP: C-reactive protein

Laboratory tests	Reference values	Measured values
White blood cells (cells/µL)	4,300-11,100	13,000
Neutrophils (%)	40-70	78%
Lymphocytes (%)	20-40	15%
Hemoglobin (g/dL)	11.5-15.5	11.2
MCV (fL)	80-100	95
Platelets (cells/µL)	150,000-400,000	138,000
Creatinine (mg/dL)	0.5-1.5	0.9
BUN (mg/dL)	8-20	14
Sodium (mmol/L)	135-145	129
Potassium (mmol/L)	3.5-5.0	3.6
Chloride (mmol/L)	98-105	97
AST (U/L)	<40	124
ALT (U/L)	<40	85
Arterial pH	7.35-7.45	7.29
Arterial PaO₂ (mmHg)	75-100	74
Arterial PaCO₂ (mmHg)	35-45	34
Arterial HCO₃⁻ (mmol/L)	22-26	19
CRP (mg/L)	<10	152
Ferritin (ng/mL)	20-300	620
Procalcitonin (ng/mL)	<0.5	0.52
D-dimer (ng/mL)	<500	1,200

**Figure 1:**
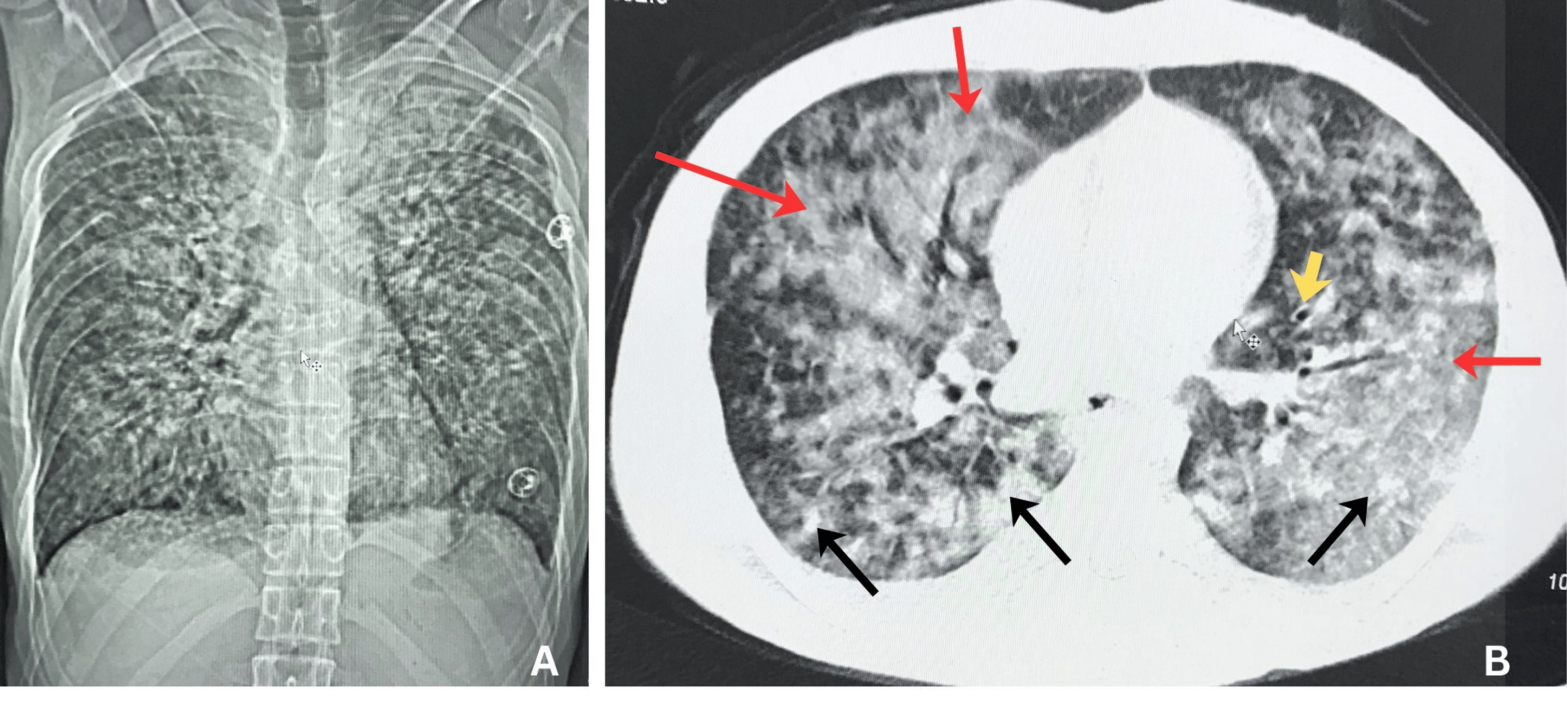
Non-contrast chest CT scout and axial views. Scout view (A) demonstrating bilateral diffuse ill-defined infiltrates and axial view (B) showing diffuse bilateral ground-glass opacities (red arrows), scattered air-space consolidations (black arrows), and peribronchial cuffing (yellow arrow).

The patient's chest radiograph showed diffuse, bilateral, ill-defined, coalescent opacities. Non-contrast chest CT showed bilateral ground-glass opacities, scattered air-space consolidations, peribronchial cuffing, limited volume of pleural effusions bilaterally, but no inflammatory micronodules were identified (Figures 1A, 1B).

Varicella pneumonia was suspected due to skin lesions and respiratory compromise. Negative results were reported for blood cultures, human immunodeficiency virus (HIV), and bacterial culture of endotracheal secretions. A varicella-zoster virus polymerase chain reaction (VZV PCR) of a bronchoalveolar lavage (BAL) specimen was positive. The diagnosis of acute respiratory distress syndrome (ARDS) caused by varicella pneumonia was maintained by the association of clinical, laboratory, chest imaging, and microbiology findings. On hospital day two, the patient was admitted to the intensive care unit (ICU) because of progressive hypoxemia and received endotracheal intubation and invasive mechanical ventilation with a lung-protective strategy. Initial treatment with intravenous (IV) acyclovir 10 mg/kg every 8 hours was started and continued for 10 days. Additionally, the patient received IV dexamethasone 20 mg once daily, IV vancomycin 1 g every 12 hours, IV cefepime 2 g every 8 hours, and IV metronidazole 500 mg every 8 hours.

As there was no evidence of bacterial infection, antibiotics were discontinued on hospital day six, and the IV dexamethasone dose was reduced to 10 mg once daily for the remainder of hospitalization. The patient was extubated on the same day after improvement in oxygenation (PaO₂/FiO₂ >300) on minimal ventilator settings (respiratory rate 14 breaths per minute, FiO₂ 30%, PIP 18 cm H₂O, and positive end-expiratory pressure {PEEP} 5 cm H₂O). Pre-extubation laboratory values are summarized in Table 2.

**Table 2:** Laboratory findings at day 6 of hospital admission. MCV: mean corpuscular volume; AST: aspartate aminotransferase; ALT: alanine aminotransferase; CRP: C-reactive protein

Laboratory tests	Reference values	Measured values
White blood cells (cells/µL)	4,300-11,100	15,200
Neutrophils (%)	40-70	82%
Lymphocytes (%)	20-40	12%
Hemoglobin (g/dL)	11.5-15.5	10.8
MCV (fL)	80-100	95
Platelets (cells/µL)	150,000-400,000	118,000
Sodium (mmol/L)	135-145	127
AST (U/L)	<40	140
ALT (U/L)	<40	92
Arterial pH	7.35-7.45	7.33
Arterial PaO₂ (mmHg)	75-100	97
Arterial PaCO₂ (mmHg)	35-45	40
Arterial HCO₃⁻ (mmol/L)	22-26	21
CRP (mg/L)	<10	65
Ferritin (ng/mL)	20-300	550
Procalcitonin (ng/mL)	<0.5	0.42
D-dimer (ng/mL)	<500	710

The patient was transferred to an isolation ward with continued antiviral therapy. With the marked improvement in dyspnea and work of breathing, the chest radiograph showed a reduction in opacification in the bilateral lung fields. Additionally, the skin rash began to crust over, with relief from pruritus. The patient was discharged on hospital day 11 after tolerating an oral diet and ambulating on ambient air. On follow-up, the patient remained well and retained his previous functional capacity.

## Discussion

VZV is a rare cause of pneumonia. It is a highly contagious disease and is acquired by inhalation of infected saliva droplets or by direct contact with skin lesions. Varicella pneumonia manifests with pulmonary symptoms, often accompanied by a vesicular skin rash [1,2]. Atypical clinical presentation, such as the absence of a skin rash, can delay diagnosis and treatment [2,4]. The patient in this case had no identifiable risk factors for varicella, such as smoking, immunosuppression, or preexisting lung disease, though his unknown vaccination status and absence of childhood chickenpox could point towards primary infection. Because of the patient's typical symptoms, clinical diagnosis of varicella pneumonia was promptly suspected, and treatment with antiviral therapy was initiated even before pursuing diagnostic tests. Clinical diagnosis may not always be as straightforward as in this case, necessitating further confirmatory laboratory tests and chest imaging.

In this case, we identified bilateral ground-glass opacities, confluent airspace consolidations, and bilateral trace pleural effusions on chest imaging. Still, no inflammatory micronodules were identified on chest CT, an unusual negative finding among patients with varicella pneumonia. Typically, chest imaging findings of varicella pneumonia show characteristic multiple nodules 1-10 mm in diameter [5-7]. The absence of characteristic nodules caused diagnostic uncertainty, as the other aforementioned radiological findings can occur in other viral pneumonias such as cytomegalovirus (CMV), influenza, and measles [2,4]. Another differential is diffuse alveolar hemorrhage, which itself can be caused by VZV [4,8].

The diagnosis was confirmed via PCR from a bronchoalveolar lavage (BAL) aspirate that is also used to exclude bacterial co-infections. In reported varicella pneumonia cases, molecular testing of skin lesion swabs, blood samples, and endotracheal aspirates was used to confirm diagnosis with high sensitivity and specificity [2,3].

This patient received treatment with acyclovir, broad-spectrum antibiotics, and corticosteroids. IV acyclovir was introduced early in the disease course based on clinical suspicion of varicella pneumonia, and was maintained for 10 days. Early antiviral therapy has been shown to reduce mortality, shorten hospital stay, and improve pulmonary outcomes in adults with VZV infection [2-4]. Empiric antibiotics were initiated at the time of intubation in accordance with local intensive care protocols, given the high risk of secondary bacterial infections in patients with viral pneumonia, and were discontinued once bacterial coinfection was excluded.

The use of corticosteroids is controversial in varicella pneumonia, but in such an infection resulting in severe hypoxemia and moderate ARDS, steroid use is justified. Corticosteroids have been shown to reduce pulmonary inflammation and improve oxygenation, though prolonged use increases the risk of opportunistic infections [2]. The early recognition, administration of antiviral therapy, and critical care support in conjunction with the patient's immunocompetency contributed to a favorable prognosis despite the development of moderate ARDS requiring mechanical ventilation.

## Conclusions

The most common disseminated varicella infection is varicella pneumonia, which can rapidly progress to life-threatening respiratory failure requiring ventilatory support even in patients with immunocompetency. The early clinical recognition and timely management of this condition improve survival outcomes. This case highlights that the absence of inflammatory nodules on chest imaging does not exclude varicella pneumonia, and molecular testing remains essential for diagnostic certainty.
